# Supramolecular solvent-based-vortex-assisted-dispersive liquid liquid microextraction of Sudan Black B in food samples prior to spectrophotometric detection

**DOI:** 10.55730/1300-0527.3661

**Published:** 2024-01-02

**Authors:** Figen EREK, Mustafa TÜZEN

**Affiliations:** 1 Department of Chemistry, Faculty of Science, Dicle University, Diyarbakır, Turkiye; 2 Department of Chemistry, Faculty of Science and Arts, Tokat Gaziosmanpaşa University, Tokat, Turkiye

**Keywords:** Preconcentration, spectrophotometric determination, Sudan Black B, supramolecular solvent, dispersive liquid liquid microextraction

## Abstract

A new, simple and rapid spectrophotometric method was developed for determination of Sudan Black B in food products by supramolecular solvent-based-vortex-assisted-dispersive liquid liquid microextraction (SUPRAS-VA-DLLME). Extraction solvent type, volume of solvent, pH, volume of THF, centrifugation time, vortex time, and sample volume were investigated as optimization parameters of the developed method. Under the optimum conditions, limit of detection and limit of quantification, preconcentration factor and enhancement factor of the developed method were calculated to be 9.01 μg L^−1^, 29.73 μg L^−1^, 20, and 55, respectively. The developed microextraction method was successfully applied to food samples for the determination of Sudan Black B.

## 1. Introduction

Sudan dyes are in the group of synthetic azo dyes [1–2]. Sudan I-V, Sudan Orange G, Sudan Black B, Sudan Red G are known as Sudan dyes [3]. These dyes are used as colorin^1^g agents in various fields of science and industry [1–2]. In many countries, the uses of Sudan dyes in food samples are prohibited due to the carcinogenicity of their reduced metabolites. According to the decisions of the European Commission, analysis reports of these dyes are required for food products [1–2,4–6]^1^. Sudan Black B (C_29_H_24_N_6_) (SBB) is an oil soluble dye widely used in biological and histological studies. SBB has a toxic effect on human liver and kidney, which is known to be carcinogenic [7]. Although usage of the dye in food products is prohibited, it has been determined that it is used in some foods. Therefore, it is necessary to develop sensitive, selective and accurate analytical methods for the detection and preconcentration of the dye from complex food matrices [4–6]^1^.

There are several techniques for separation and preconcentration of Sudan Black B in the literature. These studies were carried out with techniques such as in-line micromatrix solid-phase dispersion extraction [1], cloud point extraction [2], solvent extraction[6], magnetic solid phase extraction [7]. Microextraction methods are known as green methods compared to classical extraction methods that require a lot of toxic solvent and take a lot of time. In addition, these methods have many advantages such as cheapness, easy applicability, less organic solvent requirement and environmental friendly. In liquid phase microextraction methods, green solvents such as deep eutectic solvents, supramolecular solvents (SUPRAS), ionic liquids are widely used [8–9].

Supramolecular solvents (SUPRASs) are nonflammable, nonvolatility, inexpensive green nanostructured liquids that are formed by the combination of amphiphilic molecules. Matrix components, pH and temperature can affect the aggregation of amphiphilic molecules. In SUPRASs, amphiphilic molecules come together to form reverse micelles [10–13]. SUPRASs provide simplicity and efficiency in the extraction process with interactions such as ionic bonding, hydrogen bonding and hydrophobicity. Long-chain alcohols are used as amphiphilic molecules in alkanol-based SUPRAS microextractions. In such mixtures, alkanols spontaneously form reverse micelle aggregates. The use of a water-miscible solvent such as THF provides both the dispersion of the amphiphilic molecules and the self-assembly [14–15]. In SUPRAS-based dispersive liquid–liquid microextraction methods, vortex can be used to increase extraction efficiency and speed. Vortex time is known as a parameter that increases the interaction between the analyte and the SUPRAS phase. These methods are known as supramolecular solvent-based-vortex assisted-dispersive liquid liquid microextraction (SUPRAS-VA-DLLME [16–17]. According to our literature survey, supramolecular solvent-based-vortex-assisted-dispersive liquid liquid microextraction (SUPRAS-VA-DLLME) method has not been used for the extraction and determination of SBB in food samples.

Since the UV-visible spectrophotometer is an economical device found in all laboratories, it is very important to develop a method for the determination of SBB specrophotometrically [3]. Direct determination of SBB in water and food samples by using spectrophotometer is difficult because of low detection limit of instrument and matrix effects of contaminant ions. In order to solve these problems separation and preconcentration methods including green solvents are necessary [6].

In this study, supramolecular solvent-based-vortex assisted-dispersive liquid liquid microextraction method was developed for separation, preconcentration and determination of SBB in food samples by spectrophotometer at first time. For the optimization of the microextraction method, several parameters such as solvent type, pH, volume of solvent, volume of THF, vortex time, and centrifugation time were investigated. After the method was validated, it was successfully applied to real samples.

## 2. Materials and method

### 2.1. Apparatus

The spectra were recorded by a Carry 100 Bio UV-visible model double beam spectrophotometer. The pH measurements were made with an Toledo pH-meter. Isolab brand vortex and Hermle Z 206 A model centrifugate (max. 6000 rpm) were used for separation of the SUPRAS phase.

### 2.2. Reagents and solutions

1-dodecanol, 1-decanol, and 1-octanol were obtained from Sigma-Aldrich (St. Louis, MO, USA) for the supramolecular solvent. Methanol (HPLC grade), KF, CaCl_2_, FeCl_3_, and NiCl_2_.6H_2_O were purchased from Merck (E. Merck, Darmstadt, Germany). Sudan Black B, Eosin, Tartrazin were purchased from Fluka Chemika and Sigma Aldrich (St. Louis, MO, USA). A 100 μg mL^−1^ of stock standard solution Sudan Black B (Fluka Chemika) was prepared in methanol:water (60:40). Phosphate, acetate, ammonia buffer solutions were prepared and used for pH adjustments.

### 2.3. Real sample procedure

In this study, black rice, black bean, and three different Chili pepper were used as real samples. The real samples were taken from local markets in Diyarbakir, Turkiye. The slightly moist Chili pepper samples were dried for 1 h at 60 °C. The dried Chili pepper and other samples were weighed up to 10 g and transferred to the bakers. After adding 20 mL of methanol:water (60:40) to samples, the mixtures were sonicated for 30 min, mixed for 2 h and finally centrifuged for 10 min. Then, all samples were filtered and the developed microextraction method was applied to the filtered samples.

### 2.4. Analytical procedure

**Figure 1:**
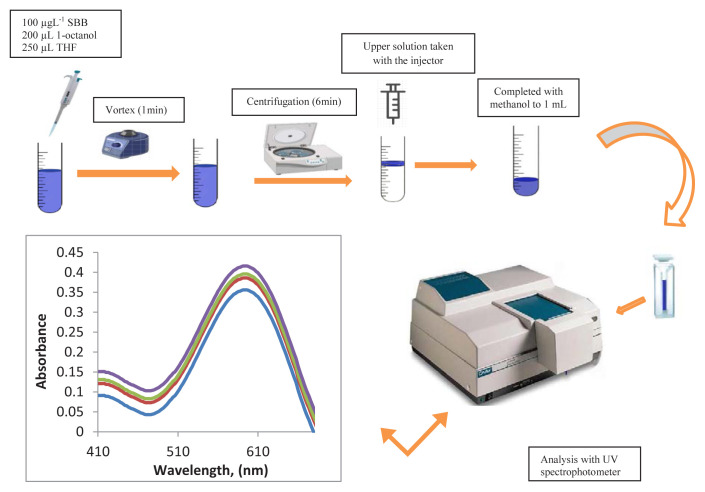
The schematic diagram of the proposed microextraction method for Sudan Black B.

Model solutions containing 20 μL of 100 μg mL^−1^ SBB solution, 4 mL of pH 6 phosphate buffer were transferred to 50 mL test tubes. The final volumes of the solutions were made up to 20 mL with distilled water. Then 0.2 mL of 1-octanol and 0.25 mL of THF were added into the solutions then vortexed for 1 min and centrifuged for 6 min. The supernatant of the samples were taken with a syringe and diluted to 1 mL with methanol. SBB concentration was determined by using UV-VIS spectrophotometer at 598 nm. The procedure is described in Figure 1.

## 3. Results and discussions

### 3.1. Effect of solvent type and volume

**Figure 2:**
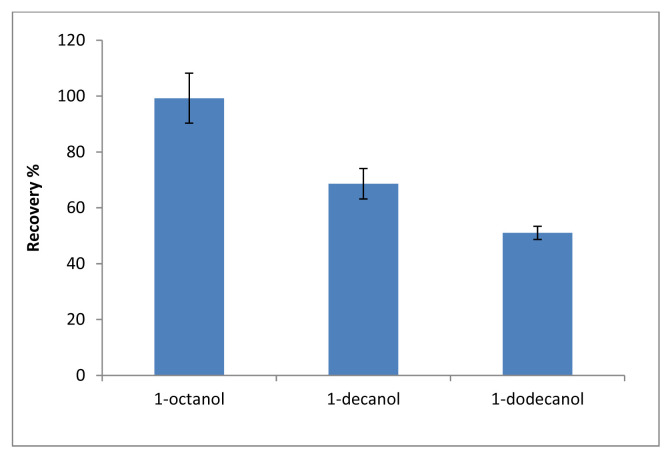
The effect of solvent type (n = 3).

**Figure 3:**
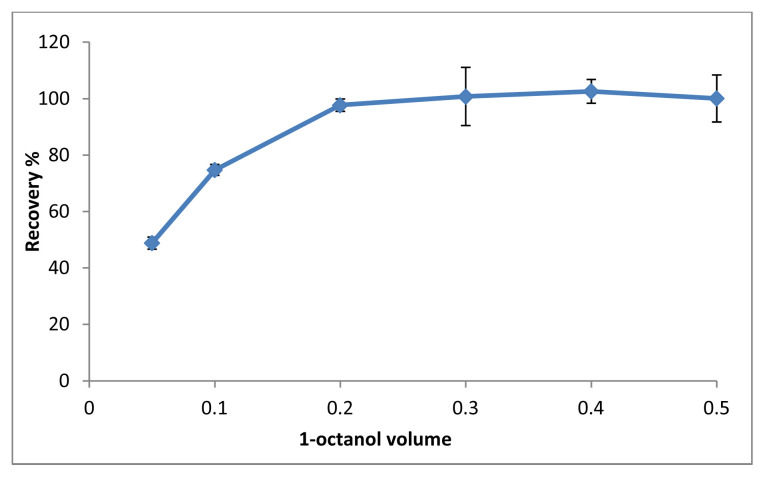
The effect of solvent volume (n = 3).

The most important step in the microextraction method is to determine the solvent type and volume. Solvents with low toxicity, low melting point near to room temperature, lower density than water can be used as SUPRAS solvents [18]. In this method, 1-octanol (mp −16 °C), 1-decanol (mp 6.4 °C) and 1-dodecanol (mp 24 °C) were investigated to obtain SUPRAS phase together with THF [18]^2^. Since 1-octanol had the highest recovery rate, it was chosen as the optimal solvent. The obtained results are shown in Figure 2. In the range of 50–500 μL, the influence of solvent volume on recovery of SBB was investigated. As shown in Figure 3, the optimal volume of 1-octanol is 200 μL.

### 3.2. Effect of pH

**Figure 4:**
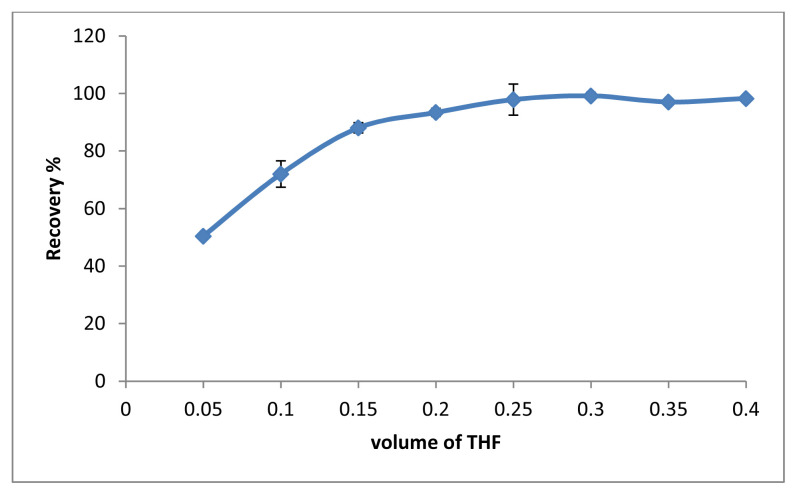
The effect of pH (n = 3).

pH has an important role for extraction efficiency in SUPRAS-DLLME. pH facilitates the transition of the analyte to the extraction phase. When the transition of the analyte to the SUPRAS phase increases, the extraction efficiency increases [8,19–21]. In order to find the optimal pH that increases the transition of SBB to the SUPRAS phase, the pH of the model solution was investigated in the range of 2–8. As shown in Figure 4, the maximum extraction efficiency was obtained at pH 6. For the subsequent studies, pH 6 was chosen as optimal pH.

### 3.3. Effect of THF volume

**Figure 5:**
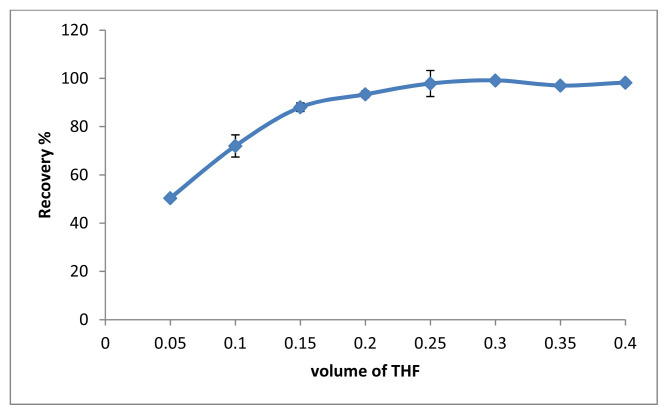
The effect of volume of THF (n = 3).

The use of a water-miscible solvent allows both dispersion of amphiphilic molecules and self-assembly in dispersive liquid liquid microextraction methods [22–23]. For this purpose, THF was used as a dispersant agent to obtain SUPRAS. In this study, the volume of THF was investigated in the range of 0.05–0.4 mL. According to the results in Figure 5, 250 μL was chosen as the optimal THF volume.

### 3.4. Effect of vortex and centrifugation time

**Figure 6:**
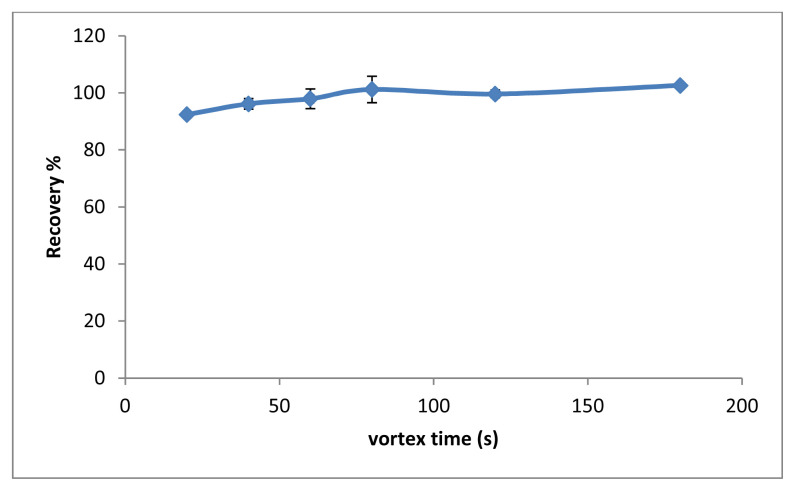
The effect of vortex time (n = 3).

The time of vortex was investigated as a parameter that increases the interaction between the analyte and the SUPRAS phase. For this purpose, the effect of vortex time was investigated in the range of 20–180s. As shown in Figure 6, the extraction efficiency was increased in the range of 20–60 and remained constant after 60 s. Considering all of these values, the optimal vortex time was determined to be 60 s.

**Figure 7:**
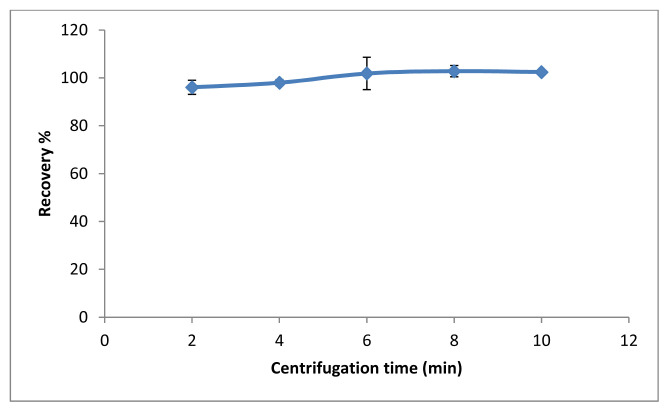
The effect of centrifugation time (n = 3).

Another factor affecting extraction efficiency is centrifugation time. Centrifugation is a step that phase separation is provided [2–3,15]. The effect of the centrifugation time on the extraction efficiency was investigated in the range of 2–10 min at max. 6000 rpm. The extraction efficiency increased until 6 min, and remained constant in the range of 6–10 min. For this reason, the optimal centrifugation time was determined to be 6 min. The results are shown in Figure 7.

### 3.5. Sample volume

To obtain a high preconcentration factor, it is necessary to determine the sample volume [24]. As seen in Table 1, the optimal sample volume is 20 mL. According to this result, the preconcentration factor was calculated to be 20 when final volume of extractant was 1 mL.

### 3.6. Matrix effect

The matrix effect is a critical point for the instrumental detection of the analyte in terms of components that can increase or decrease the analyte signal by less or more than 5% [24]. Fe^3+^, Ni^2+^, Ca^2+^, K^+^ and F^−^ ions, and dyes such as eosin, tartrazine were added into the solutions containing SBB. As seen in Table 2, the ions and dyes did not interfere in this method.

### 3.7. Analytical parameters of the developed method

The regression equation of calibration curve was linear in the range of 30–150 μg L^−1^:


$$\text{A}=0.00384\text{C}-0.0735,{\text{R}}^{2}=0.9971\;(\text{A: absorbance},\text{C: concentration of SBB }(\mu \text{g }{\text{L}}^{-1}),\text{equation after preconcentration}).$$

The RSD of the method was found to be 1.08, which calculated from the lowest concentration in the linear range with 10 replicates. In the method, limit of detection (LOD) and limit of quantitative (LOQ) were found to be 9.01 μg L^−1^ and 29.73 μg L^−1^, respectively. LOD and LOQ were calculated in the equations below:


LOD: 3 s/m and LOQ: 10 s/m[
7].

Under the optimum conditions, the preconcentration factor (PF) and enhancement factor (EF) were calculated to be 20, 55, respectively. The enhancement factor (EF) was defined as the ratio of the slope of the calibration curves after and before the developed extraction procedure [25–26]. Matrix Effects (ME) % of the method were found in the range of 4%–18%. ME% was calculated in the equation below:


ME (Matrix Effect)%=((slope of matrix-matched calibration curves/slope of standard calibration curves)-1)×100[
27]

The analytical data of the method are given in Table 3. Intraday precision studies were investigated by analyzing spiked Sudan Black B samples at 3 different concentration levels (2, 5, 10 μg/g) in one day. Interday precision studies were determined over a three-day period (N = 9). As seen in Table 4, RSD values of intraday and interday precisions are in the range of 2.05–6.20, 2.81–8.89, respectively.

### 3.8. Applying the procedure to real samples

Present SUPRAS-VA-DLLME method was successfully applied to black rice, black bean and three kinds of Chili pepper bought from local markets for the determination of SBB. Addition/recovery experiments were applied to real samples. As seen in Table 5, SBB was not detected in the samples according to the method.

### 3.9. Compared with other studies

Spectrophotometric determination of SBB using microextraction techniques has not been performed in the literature. The studies were generally carried out by chromatographic techniques. Therefore, this method was compared with chromatographic studies (Table 6). Although SUPRAS-VA-DLLME method has low EF/PF values compared to chromatographic methods, it has a wide linear range and low relative standard deviation.

## 4. Conclusions

SUPRAS-VA-based-dispersive liquid liquid microextraction method was developed for the separation, preconcentration, and determination of Sudan Black B from food samples. The developed method was easily applied to real samples. The method has several advantages such as high accuracy and precision, easy to apply without interference, low cost, using green chemicals, less solvent consumption, sensitive and selective for the determination of SBB. In the developed method, LOD, PF and EF were found 9.01 μgL^−1^, 20, 55, respectively. In the light of these results, present SUPRAS-VA-DLLME method can be easily applied to complex matrices samples for the extraction and determination of SBB.

## Figures and Tables

**Table 1:** The effect of sample volume to extraction efficiency (n = 3).

Sample volume (mL)	Recovery %

10	99.75 ± 1.08
20	101.02 ± 0.93
30	93.05 ± 0.96
40	82.80 ± 0.53

**Table 2:** The effect of interfering species to extraction efficiency (n = 3).

Interfering species	Concentration (mg L^−1^)	Recovery %
K^+^	3000	96.87 ± 1.32
Ca^2+^	250	99.78 ± 0.75
Fe^3+^	3	101.12 ± 1.34
Ni^2+^	3	100.83 ± 1.21
F^−^	3000	98.71 ± 1.23
Eosin	1	99.91 ± 0.89
Tartrazin	1	101.04 ± 1.01

**Table 3:** Summary of calibration parameters obtained using the developed microextraction approach.

Parameters microextraction	Developed
Linear equation	A* = 3.839C–0.0735
R^2^	0.9971
Linear range (μg L^−^^1^)	30–150
LOD (μg L^−^^1^)	9.01
LOQ (μgL^−^^1^)	29.73
RSD %	1.08
EF	55
PF	20
ME%**	4–18

* C:Concentration of SBB, A: Absorbance of SBB

** If matrix effect % is ≤+20% and ≥−20%, then no matrix effect is present; values >+20% and <−20% indicate signal microextraction and suppression, respectively (27).

**Table 4:** Precision of the developed procedure.

Sample		Added μg/g	Intraday precision		Interday precision	

			Recovery%	RSD%	Recovery%	RSD%
Chili pepper 1		2	100.07 ± 2.21	5.21	104.23 ± 1.05	2.83
		5	100.91 ± 2.20	3.90	101.32 ± 1.34	8.89
		10	101.04 ± 1.24	3.93	102.65 ± 2.17	2.94

Chili pepper 2		2	99.40 ± 2.85	5.21	98.76 ± 1.06	4.52
		5	99.96 ± 1.34	6.20	101.64 ± 0.92	3.66
		10	98.63 ± 2.23	4.44	100.81 ± 1.13	3.31

Chili pepper 3	2	101.21 ± 1.06	3.19	100.91 ± 0.78	2.81
	5	100.62 ± 1.71	3.64	99.18 ± 1.21	3.73
	10	100.66 ± 1.52	5.08	100.03 ± 0.52	3.01

Black rice	2	99.83 ± 2.02	6.18	101.01 ± 1.08	3.05
	5	99.04 ± 2.56	3.01	100.43 ± 2.01	4.21
	10	99.41 ± 2.06	2.05	100.82 ± 2.03	4.04

Black bean	2	100.04 ± 1.11	2.99	100.02 ± 0.97	3.12
	5	100.22 ± 1.79	5.81	101.10 ± 1.42	3.06
	10	100.54 ± 2.01	3.05	99.77 ± 1.31	3.24

* intraday RSD %, same day, N = 5; interday RSD %, three consecutive day N = 9

**Table 5:** Application of the procedure to real samples (N = 3).

Sample	Added, μg/g	Found, μg/g	Recovery, %
Chili pepper 1	–	–	–
	5	5.01	100.15 ± 0.86
	10	10.01	100.05 ± 1.02

Chili pepper 2	–	–	–
	5	5.01	100.53 ± 1.08
	10	9.98	99.41 ± 1.13

Chili pepper 3	–	–	–
	5	5.01	100.45 ± 1.12
	10	10.01	100.18 ± 0.91

Black rice	–	–	–
	5	4.98	99.15 ± 1.21
	10	9.99	99.68 ± 1.14

Black bean	–	–	–
	5	5.01	100.25 ± 1.30
	10	10.04	101.08 ± 0.98

**Table 6:** Comparison of the developed method with other studies in the literature for detemination of Sudan Black B.

Sudan dyes	Method	Enstrument	Real samples	The linear range	LOD	PF/EF	RSD%	Ref.
I–IV, Orange G, Red G, Black B	In line micromatrix solid phase dispersion extraction	HPLC	Chilli, sumac, safron, curry, paprika, turmeric	5.5–28 μgkg^−1^	1.9 μgkg^−1^	–	–	[1]
I–IV, Orange G, Red G, Red 7B, Black B	Cloud point extraction	LC/MS	Wine, sauce, chilli	0.3–25 mgkg^−^^1^	0.03–0.3 mgkg^−^^1^	–	<20	[2]
Black B	aceton extraction	Raman spectroscopy	black rice	0.05–2 mgL^−^^1^	–	–	<5	[7]
I–IV, Orange G, Red G, Black B	Magnetic solid phase microextractions	HPLC	chilli	3–60 ngmL^−^^1^	0.16 ngmL^−^^1^	167	1.8	[8]
I–IV, Orange G, Red G, Red 7B, Black B	single-step extraction	LC/tandem-MS	Sauce, cotton candy, pickle	1–100 ngmL^−^^1^	3.2, 5, 2.7 μgkg^−1^	–	–	[28]
Black B	VA-SUPRAS-LPME	UV-VIS	Chilli, black rice, black bean	30–150 μgL^−^^1^	9.01 μgL^−^^1^	20/55	1.08	This study

* HPLC: High performance liquid chromatography, LC/MS: Liquid chromatography/mass spectrometer, UV-VIS: Ultraviole-Visible spectrophotometer, VA-SUPRASS-LLME: Vortex-assisted supra molecular solvent based liquid-liquid microextraction

## Footnotes

1 European Union (2004). Rapid Alert System For Food And Feed (RASFF) [online]. http://ec.europa.eu/food/food/rapidalert/report2004_en.pdf [accessed 03 January 2022].

2 Inchem (2022). 1-Octanol. https://inchem.org/documents/icsc/icsc/eics1030.htm [accessed 14 January 2023].
